# Genetic Variation in Healthy Oldest-Old

**DOI:** 10.1371/journal.pone.0006641

**Published:** 2009-08-14

**Authors:** Julius Halaschek-Wiener, Mahsa Amirabbasi-Beik, Nasim Monfared, Markus Pieczyk, Christian Sailer, Anita Kollar, Ruth Thomas, Georgios Agalaridis, So Yamada, Lisa Oliveira, Jennifer A. Collins, Graydon Meneilly, Marco A. Marra, Kenneth M. Madden, Nhu D. Le, Joseph M. Connors, Angela R. Brooks-Wilson

**Affiliations:** 1 Canada's Michael Smith Genome Sciences Centre, British Columbia Cancer Agency, Vancouver, British Columbia, Canada; 2 Institute of Plant Biology, University of Zürich, Zürich, Switzerland; 3 Department of Medicine, University of British Columbia, Vancouver, British Columbia, Canada; 4 Cancer Control Research, British Columbia Cancer Agency, Vancouver, British Columbia, Canada; 5 Centre for Lymphoid Cancer, British Columbia Cancer Agency, Vancouver, British Columbia, Canada; 6 Department of Biomedical Physiology and Kinesiology, Simon Fraser University, Burnaby, British Columbia, Canada; Brunel University, United Kingdom

## Abstract

Individuals who live to 85 and beyond without developing major age-related diseases may achieve this, in part, by lacking disease susceptibility factors, or by possessing resistance factors that enhance their ability to avoid disease and prolong lifespan. Healthy aging is a complex phenotype likely to be affected by both genetic and environmental factors. We sequenced 24 candidate healthy aging genes in DNA samples from 47 healthy individuals aged eighty-five years or older (the ‘oldest-old’), to characterize genetic variation that is present in this exceptional group. These healthy seniors were never diagnosed with cancer, cardiovascular disease, pulmonary disease, diabetes, or Alzheimer disease. We re-sequenced all exons, intron-exon boundaries and selected conserved non-coding sequences of candidate genes involved in aging-related processes, including dietary restriction (*PPARG, PPARGC1A, SIRT1, SIRT3, UCP2, UCP3*), metabolism (*IGF1R, APOB, SCD*), autophagy (*BECN1, FRAP1*), stem cell activation (*NOTCH1, DLL1*), tumor suppression (*TP53, CDKN2A, ING1*), DNA methylation (*TRDMT1, DNMT3A, DNMT3B*) Progeria syndromes (*LMNA, ZMPSTE24, KL*) and stress response (*CRYAB, HSPB2*). We detected 935 variants, including 848 single nucleotide polymorphisms (SNPs) and 87 insertion or deletions; 41% (385) were not recorded in dbSNP. This study is the first to present a comprehensive analysis of genetic variation in aging-related candidate genes in healthy oldest-old. These variants and especially our novel polymorphisms are valuable resources to test for genetic association in models of disease susceptibility or resistance. In addition, we propose an innovative tagSNP selection strategy that combines variants identified through gene re-sequencing- and HapMap-derived SNPs.

## Introduction

Aging is a universal trait shared among most, if not all organisms [1]–[3]. The rate and extent of aging, however, varies substantially between species. The observed maximum human lifespan of 122 years (Jeanne Calment, France) is surpassed only by rougheye rockfish, red sea urchins, bowhead whales, and the Galapagos land tortoise (all 150–200 years) [1], [4]–[6]. A variety of theories of why and how organisms age have been proposed, including oxidative damage, telomere shortening, accumulation of mutations and others (reviewed in [7]), but the contribution of individual genes and variation within these genes is still under investigation.

Molecular aging research has advanced substantially in recent years through genomics and proteomics approaches, particularly their application to understanding aging in various model organisms. High-throughput screens for mutations that extend lifespan in *Saccharomyces cerevisiae, Caenorhabditis elegans*, and *Drosophila melanogaster* were successful in highlighting that deletion or attenuation of single genes can result in substantial lifespan extensions [8]. For example, the most intensively studied aging-related gene in *C. elegans* is *daf-2*, an insulin/IGF receptor [9], [10]. Reduction of *daf-2* signaling in mutant worms leads to a doubling of mean lifespan [11]. Mutations in the *daf-2* homologs of *Drosophila* and mice showed an 80% and 30% increase in lifespan, respectively [12], [13].

Human lifespan is also determined in part by genetic factors. The heritability of human longevity is estimated as approximately 25% [14]. This estimate is also supported by a study of the entire population of Iceland [15]. In addition, it was shown that siblings of centenarians have a 4-fold greater probability of surviving to the age of 91 [16]. A study of families with long-lived siblings localized a longevity locus to a region on chromosome 4 [17], [18].

In contrast to genetic loci related to extreme longevity, mutations in single genes underlie several human premature aging syndromes. Among these, Werner (OMIM: 277700), Bloom (OMIM: 210900) and Hutchinson-Gilford Progeria (HGP; OMIM: 176670) syndromes are segmental accelerated aging syndromes. These severe conditions are caused by mutations in DNA helicases in Werner and Bloom syndromes and in Lamin A in HGP patients [19], [20]. The consequences of these mutations are impaired DNA repair/maintenance or nuclear instability, which affect cell survival and tissue homeostasis [21]. This evidence together argues for specific ‘aging genes’ that may represent key components of pathways which when modulated results in pro- or anti-aging effects. Variation within such genes may be a factor in the inter-individual heterogeneity of human lifespan.

As a first step towards investigating the effects of genetic variation in aging-related genes on human lifespan and health, we characterized genetic variation in healthy oldest-old. To assess the genetic variation in a selection of aging-related candidate genes we have re-sequenced 24 genes in healthy seniors 85 years old or older. Candidate genes were selected either through gene expression analysis of long-lived *C. elegans daf-2* mutants [10] or literature reports. Genes previously identified as differentially expressed in long-lived *daf-2* mutants compared to wild type worms included genes involved in metabolism (*IGF1R* (GeneID: 3480), *SCD* (GeneID: 6319), *APOB* (GeneID: 338)) and stress response (*CRYAB* (GeneID: 1410), *HSPB2* (GeneID: 3316)). Response to dietary restriction (DR) is suggested to be an evolutionary conserved mechanism that enhances survival in adverse environmental condition but also extends lifespan [22]. Key genes of dietary restriction-mediated lifespan extension have been identified in animal studies and include sirtuins (*SIRT1* (GeneID: 23411), *SIRT3* (GeneID: 23410)), uncoupling proteins (*UCP2* (GeneID: 7351), *UCP3* (GeneID: 7352)), *PPARG* (GeneID: 5468) and *PPARGC1A* (GeneID: 10891). Additional candidate gene categories chosen, included autophagy (*BECN1* (GeneID: 8678), *FRAP1* (GeneID: 2475)), tumor suppression (*TP53* (GeneID: 7157), *CDKN2A* (GeneID: 1029), *ING1* (GeneID: 601566)), DNA methylation (*TRDMT1* (GeneID: 1787), *DNMT3A* (GeneID: 1788), *DNMT3B* (GeneID: 1789)), stem cell activation (*NOTCH1* (GeneID: 4851), *DLL1* (GeneID: 28514)), and Progeria syndromes (*LMNA* (GeneID: 4000), *ZMPSTE24* (GeneID: 10269), *KL* (GeneID: 9365)). References for all candidate genes are provided in **Table 1**.

**Table 1:** Candidate genes, relevance to aging and biological function.

Relevance to Aging	Gene Name (*H. sapiens*/*C. elegans*)	Biological Function	Reference
Gene Expression Study	IGF1R/*daf-2*	growth factor/IGF-1 signaling	[10]
(*C. elegans daf-2*)	SCD/*fat* - gene family	lipid metabolism, Stearoyl-CoA desaturase	
	APOB/*vit* - gene family	lipid metabolism, low density lipoproteins	
	CRYAB/*hsp-12 and hsp-16*	small heat shock protein	
	HSPB2/*hsp-25*	small heat shock protein	
Dietary Restriction	SIRT1	NAD-depended deacetylase	[48]
	SIRT3	mitochondrial respiration, ROS production	[49]
	UCP2	uncoupling protein, ROS production	[50]
	UCP3	uncoupling protein, ROS production	[51]
	PPARG	key regulator of white adipose tissue	[52]
	PPARGC1A	mitochondrial biogenesis	[53]
Autophagy	FRAP1	environmental sensor, general metabolism	[54]
	BECN1	key regulator of autophagy	[55]
Stem Cell Activation	NOTCH1	muscle satellite cell activation	[56]
	DLL1	ligand of NOTCH1	
Progeria Syndrome	LMNA	mutated in HGPS	[19]
	ZMPSTE24	posttranslationally modifies LMNA protein	[57]
	KL	preamature aging in mouse	[58]
Tumor Suppression	TP53	tumor suppression, cell cycle control	[59]
	ING1	tumor suppression, apoptosis	[60]
	CDKN2A	tumor suppression, cellular senescence	[61]
DNA Methylation	TRDMT1	DNA/RNA methytransferase	[62]
	DNMT3A	de novo DNA methyltransferase	[63]
	DNMT3B	de novo DNA methyltransferase	[27]

ROS - reactive oxygen species (free radicals)

HGPS - Hutchinson-Gilford Progeria Syndrome

Genes from these various biological pathways are functionally interconnected; for instance, DR affects most, if not all, of the other categories of genes. DR reduces cancer risk in model organisms [23], induces autophagy [24], changes DNA methylation [25] and even ameliorates loss of stem cell function with age [26]. Other examples of inter-pathway connections are DNA methylation and tumor suppression [27] as well as Progeria syndromes and stem cell activity [21].

In addition to characterizing the extent of genetic variation in these aging-related candidate genes, our second goal was to establish tagSNP sets that incorporated both genetic information from a reference population, as well as specific variants discovered in our exceptional healthy oldest-old. This combined approach benefits from valuable information generated by the HapMap project [28] as well as incorporating rare alleles, which in aggregate may have major contributions to susceptibility or resistance to disease [29].

To characterize the extent of genetic variation in 24 candidate ‘healthy aging’ genes we have re-sequenced the exons, intron-exon boundaries, 1500 bp upstream regions and conserved sequences in 47 healthy oldest-old. We present a catalogue of genetic variation in aging-related genes and highlight the value of gene re-sequencing for identifying SNPs for testing in genetic association studies.

## Results

### Variant Discovery by Gene Re-sequencing

**Table 1** summarizes the 24 candidate healthy aging genes, their biological function, and relevance to aging or longevity. For variant detection, we used blood DNA from 47 healthy oldest-old (mean age 89 years, median age 88 years). These individuals have never been diagnosed with cancer, cardiovascular disease, diabetes, Alzheimer disease or major pulmonary disease.

For each candidate gene, we bi-directionally re-sequenced all exons (including exons of known alternative transcripts), 5′ and 3′ untranslated regions (UTRs), all intron-exon junctions, 1500 bp upstream (including the core promoter) and selected conserved non-coding sequences (CNS). Our criteria for a CNS was a minimum of 70% conservation over at least 100 bp. **Table 2** summarizes amplicon sequencing and variant discovery. Per individual, we sequenced 716 amplicons and ∼360 Kb of DNA. Overall, we generated and analyzed ∼35 million high quality (Phred 20) DNA bases. We detected 935 variants (on average one every 400 bp), of which 550 (59%) are represented in dbSNP (build 126) and 385 (41%) were novel. 87 (9%) are insertions or deletions. The average minor allele frequency (MAF) is 15%. There is a marked difference in the average MAF of markers represented in dbSNP and ones that are not, 21% and 6% respectively. 72% (398/550) of variants that are in dbSNP have a MAF of ≥5%, whereas only 22% (84/385) of novel variants have a MAF of 5% or greater. Despite a MAF of 19% for all insertion/deletion variants, only 30% (26/87) have an entry in dbSNP. BECN1 and DNMT3A show the lowest degree of variation (1.1 variant/1000 bps), whereas SIRT3 and TRDMT1 have the highest; 5.5 and 5.1 variants/1000 bps, respectively. **Supplemental online Table S1** lists the 935 variants discovered through candidate gene re-sequencing, their genomic location, flanking sequences, dbSNP rs number, nucleotide difference, codon/amino acid difference, and MAF.

**Table 2:** Amplicon sequencing and variant discovery.

Gene Name	Number of Amplicons (Exon, CNS^a^)	Kb Sequenced	Number of Variants	Variants per 1000 bp	Number of Novel Variants (%)^b^	Insertion or Deletion
**IGF1R**	52 (29, 23)	20	68	3.4	33 (49)	9
**SCD**	26 (20, 6)	12	30	2.5	12 (40)	6
**APOB**	75 (56, 19)	33	57	1.7	21 (37)	1
**CRYAB**	10 (3, 7)	4	8	1.8	3 (38)	0
**HSPB2**	5 (4, 1)	2	4	1.8	1 (25)	0
**SIRT1**	21 (18, 3)	11	30	2.7	17 (57)	7
**SIRT3**	14 (10, 4)	7	40	5.5	13 (33)	3
**UCP2**	14 (8, 6)	7	19	2.7	12 (63)	3
**UCP3**	18 (10, 8)	10	38	3.9	17 (45)	4
**PPARG**	21 (11, 10)	12	33	2.8	11 (33)	3
**PPARGC1A**	36 (23, 13)	20	50	2.6	28 (56)	6
**FRAP1**	74 (64, 10)	39	65	1.7	22 (34)	2
**BECN1**	19 (10, 9)	9	10	1.1	8 (80)	3
**NOTCH1**	50 (37, 13)	28	117	4.1	46 (39)	4
**DLL1**	22 (12, 10)	11	28	2.5	13 (46)	1
**LMNA**	26 (18, 8)	13	30	2.2	9 (30)	4
**ZMPSTE24**	22 (18, 4)	11	15	1.3	4 (27)	0
**KL**	32 (22, 10)	17	41	2.5	14 (34)	4
**TP53**	26 (16, 10)	7	36	4.9	13 (36)	3
**ING1**	22 (19, 3)	11	26	2.3	13 (50)	7
**CDKN2A**	16 (14, 2)	11	15	1.4	8 (53)	0
**TRDMT1**	29 (25, 4)	16	80	5.1	26 (33)	9
**DNMT3A**	49 (38, 11)	28	31	1.1	21 (68)	3
**DNMT3B**	37 (30, 7)	20	64	3.2	20 (31)	5
**Total**	**716 (515, 201)**	**360**	**935**	**avg. 2.7**	**385 (41%)**	**87 (9%)**

a conserved non-coding sequences (CNS) and 1500 bp upstream.

b dbSNP (build 126), Kb - kilobase pairs.

**Table 3** summarizes the locations of these genetic variants within each gene. The highest numbers of variants are within introns (353). The second most abundant group of variants is found in CNS (317). These include highly conserved non-coding sequences that were chosen through phylogenetic footprinting (within±15 kb of the candidate genes), and 1500 bp upstream of the transcriptional start sites. Furthermore, we found 128 variants in 5′ and 3′ UTRs and seven within 6 bp of exon-intron junctions (splice site variants). Within the coding region of our candidate genes, we found 54 non-synonymous and 76 synonymous variants. All of these are SNPs, with one exception; a Leu-Leu-Ala deletion in exon 1 of APOB. The 80 coding variants that are represented in dbSNP are, on average, more common (average MAF 19%) than our 50 novel coding SNPs (average MAF 1.6%).

**Table 3:** Genomic location of variants.

Gene Name	Non-Synonymous	Synonymous	UTR^a^	Flanking, CNS^b^	Splice Site^c^	Intron	TOTAL
**IGF1R**	2	8	10	35	1	12	68
**SCD**	1	0	14	10	0	5	30
**APOB**	19	8	2	24	0	4	57
**CRYAB**	1	1	0	3	1	2	8
**HSPB2**	1	1	0	2	0	0	4
**SIRT1**	2	2	5	7	1	13	30
**SIRT3**	4	2	6	8	1	19	40
**UCP2**	1	1	8	3	0	6	19
**UCP3**	2	2	5	18	0	11	38
**PPARG**	1	1	0	9	0	22	33
**PPARGC1A**	3	3	12	25	0	7	50
**FRAP1**	0	6	5	14	0	40	65
**BECN1**	0	0	1	5	0	4	10
**NOTCH1**	5	13	9	13	1	76	117
**DLL1**	1	3	3	14	0	7	28
**LMNA**	0	5	3	12	0	10	30
**ZMPSTE24**	0	1	2	4	0	8	15
**KL**	3	6	3	18	0	11	41
**TP53**	1	3	3	19	0	10	36
**ING1**	1	1	13	8	0	3	26
**CDKN2A**	3	0	4	6	0	2	15
**TRDMT1**	2	4	12	37	1	24	80
**DNMT3A**	0	2	2	10	0	17	31
**DNMT3B**	1	3	6	13	1	40	64
**Total**	**54**	**76**	**128**	**317**	**7**	**353**	**935**

a UTR - 5′ and 3′ untranslated regions.

b Flanking, CNS includes 1500 bp upstream of genes and 3′ gene flanking sequence. CNS = conserved non-coding sequences.

c within 6 bp from the exon-intron junction.

### TagSNP Selection: Combining Public HapMap and Private Re-sequencing Variants

**Figure 1:**
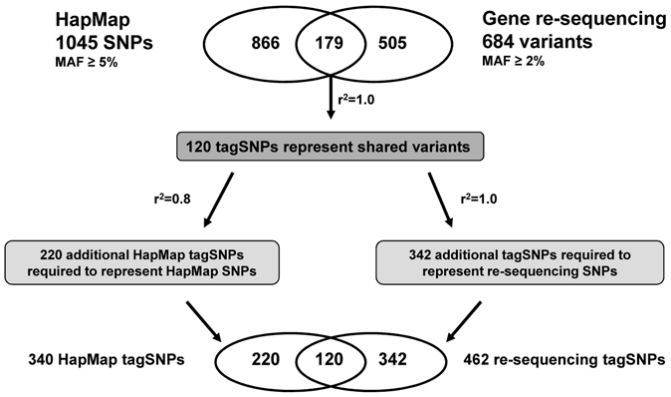
TagSNP selection strategy. HapMap genotypes for European individuals were obtained from the HapMap database. For each candidate gene, SNPs within the gene region±10 Kb were included. A MAF ≥5% and an r^2^ = 0.8 were used for tagSNPs selection using Haploview. Variants with a MAF ≥2% were analyzed in the gene re-sequencing set, with r^2^ = 1.0. Using a two-stage approach, we selected 682 tagSNPs that represent 1550 non-redundant variants from gene re-sequencing and HapMap data sets. tagSNPs (120) representing the 179 shared variants found in both data sets were determined in the gene re-sequencing set. CNS = conserved non-coding sequences.

To minimize the number of variants that have to be genotyped in association studies, we devised an innovative tagSNP selection protocol outlined in **Figure 1**. This protocol is designed to incorporate both common and less common variants. Because variants discovered in healthy oldest-old represent potential healthy aging alleles, we include SNPs with MAF ≥2% that were found in re-sequencing. This was equivalent to including variants that had been observed at least twice, but excluded ‘singletons’ that had been observed only once. To pick tagSNPs representing SNPs found by re-sequencing, we set a high threshold of r^2^ = 1.0, since we did not want to leave out any SNPs from this unique source. To cover genetic variation in the introns and gene proximity we also included HapMap SNPs for the European ancestry population (CEU) chosen based on genomic regions of candidate genes±10 Kb. MAF ≥5% and r^2^ = 0.8 were used to choose representative tagSNPs from among these known SNPs.

To apply our SNP selection method, first a set of 179 SNPs found in both our re-sequencing SNP set and in HapMap was identified, and 120 tagSNPs were picked to represent them with MAF ≥2% and r^2^ = 1.0 in the re-sequencing set. These SNPs were then prioritized in the selection of additional tagSNPs to represent the set of HapMap SNPs in the region that had minimum MAF = 5%, at r^2^ = 0.8. They were also prioritized in the selection of additional tagSNPs to represent the other re-sequencing SNPs, with MAF ≥2%, at r^2^ = 1.0.

**Table 4** summarizes the results of tagSNP selection using this method, including the number of variants available per gene and the resulting number of tagSNPs. 340 tagSNPs (32%) represent 1045 HapMap SNPs, whereas 462 tagSNPs (67%) represent 684 gene re-sequencing variants. Our approach selected 682 tagSNPs that represent 1550 variants, representing a 56% reduction of variants that need to be genotyped to represent the entire set. The majority of tagSNPs are actual SNPs, whereas, exclusively in the re-sequencing set, a few ‘tagSNPs’ refer to insertions or deletions.

**Table 4:** tagSNP selection; HapMap versus candidate gene re-sequencing.

Gene Name	Number of variants					Number of tagSNPs				
	Sequencing Variants^a^	Shared Variants^b^	HapMap Variants^c^	Total non-redundant Variants	Shared (%)	Sequencing tagSNPs^d^	Shared tagSNPs	HapMap tagSNPs^e^	Total non-redundant tagSNPs	Shared (%)
**IGF1R**	48	2	122	168	1	44	2	36	78	3
**SCD**	25	7	31	49	14	14	6	12	20	30
**APOB**	37	11	38	64	17	31	10	16	37	27
**CRYAB/HSPB2** #	12	4	8	16	25	10	3	3	10	30
**SIRT1**	16	5	27	38	13	10	3	5	12	25
**SIRT3**	32	11	37	58	19	16	5	13	24	21
**UCP2**	13	4	17	26	15	7	2	6	11	18
**UCP3**	29	7	23	45	16	18	6	8	20	30
**PPARG**	23	9	117	131	7	19	6	24	37	16
**PPARGC1A**	30	12	112	130	9	24	11	51	64	17
**FRAP1**	45	9	43	79	11	13	5	7	15	33
**BECN1**	11	0	2	13	0	11	0	2	13	0
**NOTCH1**	93	19	38	112	17	75	18	34	91	20
**DLL1**	17	3	14	28	11	13	3	13	23	13
**LMNA**	23	6	18	35	17	11	3	6	14	21
**ZMPSTE24**	11	7	23	27	26	9	6	10	13	46
**KL**	32	13	101	120	11	22	5	22	39	13
**TP53**	22	1	14	35	3	15	1	8	22	5
**ING1**	17	3	13	27	11	14	1	3	16	6
**CDKN2A**	15	2	23	36	6	14	2	9	21	10
**TRDMT1**	64	24	124	164	15	29	9	12	32	28
**DNMT3A**	18	4	51	65	6	15	3	23	35	9
**DNMT3B**	51	16	49	84	19	28	10	17	35	29
**Total**	**684**	**179**	**1045**	**1550**	**12%**	**462**	**120**	**340**	**682**	**18%**

a MAF ≥2%.

b variants observed in the re-sequencing and HapMap data sets.

c MAF ≥5%.

d r2 = 1.0.

e r2 = 0.8.

# CRYAB/HSPB2 are adjacent to each other on chromosome 11 and were analyzed together.

Of note is the low degree of overlap between HapMap and gene re-sequencing variants. For all 24 candidate genes analyzed, only 12% of variants (179/1550) are shared between available HapMap SNPs and sequencing data generated from our healthy oldest-old. As the LD structure of variants only present in one set cannot be inferred in the other set, HapMap and candidate gene re-sequencing variants have to be treated, for LD-based tagSNP selection, as different data sets. Prioritizing variants found in both sets in our tagSNP selection method, however, increased the number of overlapping SNPs in the tagSNP set to 18% (120/682) **(** **Figure 1** **)**.

We conclude from this analysis that if only SNPs available through the HapMap project had been chosen to represent the regions sequenced in our candidate genes, we would only have represented 26% of the variants (179/684) that are actually present in our study population **(** **Figure 1** **and** **Table 4** **)**. Subsequently, tagSNPs chosen using this combined method were genotyped in 493 healthy oldest old and 439 random individuals aged 40–50, using the Illumina GoldenGate method (data not shown). Only 7 out of 245 (2.4%) private re-sequencing tagSNPs were represented by 297 HapMap tagSNPs at r^2^ = 0.8.

## Discussion

Besides genes that have been shown to affect lifespan in animal models, a limited number of genetic variants have been reported to be associated with long life in humans. These studies mainly evaluated genetic variation linked to extreme human life spans (e.g. centenarians) without focusing specifically on health. Such genes include *APOE* (GeneID: 348) [30], [31], *CETP* (GeneID: 1071) [32], Interleukin 6 (GeneID: 3569) [33], [34], Interleukin 10 (GeneID: 3586) [35], [36], *PON1* (GeneID: 5444) [37], FOXO3A (GeneID: 2309) [38], [39] and *SIRT3* [40]. Controversy exists regarding the contribution of these and other gene variants to aging and longevity, because replication studies in different populations, as for replication studies in complex diseases, more often than not fail to confirm the initially reported associations. For instance, the common polymorphism I405V in *CETP* that was associated with longevity in Ashkenazi Jewish centenarians was not confirmed in an Italian replication study [41]. A comprehensive summary of genetic variants that have been tested for association with human aging/longevity can be found at http://genomics.senescence.info/genes. Almost exclusively, these studies tested single variants in candidate genes without surveying the whole gene in a more comprehensive manner.

For a limited number of genes, including APOE, FOXO3A, and PON1, association of specific variants with aging/longevity has already been established [30], [31], [37]–[39]. These associations, however, only account for a fraction of the genetic contribution to aging and longevity. Our candidate gene choice reflects the need to assess genetic variation in a broader spectrum of genes that affect aging-related biological mechanisms and pathways, particularly in animal models. Although it is plausible that additional ‘causal’ variants exist in these documented aging-associated genes, we focused on an independent set of genes to generate genetic variation data for use in association studies.

Large-scale sequencing efforts will be necessary to construct a complete picture of genetic contributions to aging and other complex phenotypes. Although advances in DNA sequencing technologies will ultimately provide sequence information of all exons and whole genomes, it will take time until comprehensive genomic information will be available for large cohorts of long-lived individuals. Until then, targeted re-sequencing studies, as presented here, will add value to genetic epidemiology studies.

The common variant common disease hypothesis proposes that genetic susceptibility to common conditions and diseases like hypertension and diabetes is largely due to alleles that have moderate frequency in the population [42]. The ‘rare variant hypothesis’ in contrast, argues that a significant proportion of inherited susceptibility to relatively common chronic diseases is due to the cumulative effects of many low frequency dominantly and independently acting variants of a variety of different genes, and that each of these variants confers a moderate increase in relative disease risk [29]. For many diseases, it is not yet clear which of these hypotheses, or both, will be applicable. Current genome-wide association studies (GWAS) are capable of testing for association with many, even a million, relatively common SNPs, but do not comprehensively test for association with rare variants. Current studies may therefore neglect the effects of this important set of genetic variants [29]. Healthy aging is an uncommon phenotype (we estimate that <12% of individuals born will go on to achieve our definition of healthy aging). Rare variants, with more substantial genetic effects, are generally more important in rare disorders than common ones [29]. We reason that multiple rare variants could in theory play a role in the healthy aging phenotype.

We have re-sequenced aging-related candidate genes to systematically detect common *and* rare variants that potentially contribute to healthy aging and disease-resistance. This set of 935 variants (summarized in **supplemental online Table S1**) will provide a valuable resource for the bio-gerontological as well as the biomedical communities, the more so because rare variants and particularly insertions and deletions are underrepresented in dbSNP and HapMap [43]. Some rare missense variants or variants in the promoter or other gene regulatory regions may have effects on gene expression [29]. In our study, 201 (of 716) amplicons covered conserved nucleotide sequence regions. Variants in these regions (including UTRs) accounted for 48% of all variants (445/935), providing a substantial data set for functional studies of these genes.

Current genotyping studies, regardless of phenotype, typically rely on common ethnicity-specific HapMap tagSNPs to represent common variation in targeted regions (candidate genes) or within whole genomes (GWAS). We reason that healthy aging and longevity are unlikely to be due solely to the presence of a small number of common variants. This desirable phenotype may in part be due to absence of specific disease-causing alleles, as well as presence of favorable combinations of other alleles. For this reason, limiting association studies of healthy aging or longevity to testing common SNPs may be unsuccessful, or at best incompletely successful. Ultimately, more sophisticated analyses enabled by full genome sequencing will allow the assessment of both common and rare variants. In the meantime, a relatively cost effective approach for candidate gene-based analyses is to perform SNP discovery in cases with the phenotype of interest, for later comparison to appropriate controls. In this study, we not only establish a catalog of genetic variation in genes relevant to aging in healthy oldest old, we also use this data to ask whether current public SNP resources can represent this deeper variation. We find that, while HapMap tagSNPs are known to be very useful for representing common variants, they do not adequately represent uncommon variants for studies of uncommon phenotypes of interest like healthy aging.

The variants reported here, especially our novel polymorphisms, can be taken forward by the aging research community, as well as by investigators who study these genes with regard to diseases, to test for genetic association in relevant populations. In addition, our data informs the study designs of the future, by helping to justify larger scale next-generation sequencing for enabling more comprehensive comparisons of groups of cases and controls.

Our approach aims to provide deeper coverage and enable analysis of both common and rare variants, without unnecessarily increasing genotyping costs and effort. It involves selecting a minimal set of tagSNPs that represent two or more independent sets of SNPs from each candidate gene. A final tagSNP list generated with our combined selection method is limited to the ethnicity it was generated for. TagSNP selection for other ethnicities would require separate analyses, ideally using HapMap data from that population and re-sequencing data from the same population. Transferability in between study populations of the same ethnicity (e.g. oldest-old, centenarians, or super-centenarians) is feasible but would solely depend on the presence of the private re-sequencing variants amongst these populations.

Our tagSNP selection strategy uses Tagger, a well-documented tool for the selection and evaluation of tagSNPs from genotype data [44]. The main value of our tagSNP selection process is based on the fact that future targeted genotyping projects, as opposed to whole genome SNP scans, will combine variation information from public (HapMap) and private sources potentially derived from next-generation sequencing of individuals with phenotypes of interest. Our study design is capable of finding effects due to rare variants if subsequently tested in a large enough case/control resource. The strategy in which regional re-sequencing is done only after a region of interest is identified through a HapMap-based strategy, will generally not detect the effects of rare variants.

Our tagSNP analysis showed that only 19% (179/935, Table 2 and Figure 1) of variants seen in the re-sequencing set are also represented in HapMap. This discrepancy is based on the facts that HapMap mainly supports common variants (MAF ≥5%) and that those variants were chosen to distribute relatively uniformly across genomic regions. In our re-sequencing data, 28% (264/935) of all variants are singletons, emphasizing the abundance of private variants within individuals. Contributions of these rare variants to individual disease risk cannot be evaluated solely using HapMap tagSNPs.

In genotype data for the combined tagSNP set, generated in 493 healthy oldest old and 439 random individuals aged 40–50, only 7 out of 245 (2.4%) private re-sequencing tagSNPs were represented by 297 HapMap tagSNPs at r^2^ = 0.8. This shows that HapMap tagSNPs generally do not adequately represent, private re-sequencing SNPs. This analysis highlights a major challenge for genetic association studies. Using only HapMap SNPs, effects due to uncommon variants would often be missed.

Healthy oldest-old rather than centenarians or healthy centenarians have been chosen for this study based on demographic data, which suggests that in western countries less than 36% of individuals live up to 85 years and that only one third of these will do so in good health (∼12% overall) [45]. Hence, healthy oldest-old are uncommon in the population, but not as rare as centenarians (1 per 3300 people in the US). We have collected lifestyle, education and other information for the entire collection of healthy oldest-old (550 individuals, of which the 47 individuals sequenced are a subset) and also for 550 controls for use in future association study, to be able to control for major lifestyle and socio-economic factors in future association studies.

Our study is the first to present a comprehensive analysis of genetic variation in aging-related candidate genes in the healthy oldest-old. Genetic association studies of aging and longevity to date have relied mainly on known variants or on common variants from dbSNP and HapMap as detailed genetic variation maps of aging-related genes in individuals of advanced age are not yet available. Testing these variants in case-control studies or families with a history of long-lived individuals can greatly assist the search for genetic factors that contribute to successful and healthy aging and longevity.

## Materials and Methods

This study was approved by the joint Clinical Research Ethics Board of the British Columbia Cancer Agency and the University of British Columbia. All subjects gave written informed consent.

### Study Participants

Subjects were recruited between January 2004 and August 2007 in the Greater Vancouver Regional District in British Columbia, Canada. Participants were 85 years or older at the time of recruitment and reported that they had never been diagnosed with cancer, cardiovascular disease, diabetes, major pulmonary disease, or Alzheimer disease. We conducted detailed questionnaires about their personal and family medical history, medication and supplements. We also took blood pressure measurements, assessed their body mass index, smoking habits, alcohol consumption, physical exercise, stress history, and educational and occupational background. Standard geriatric tests to assess memory, cognition, psychological status, and mobility of study subjects were performed. These included the Folstein Mini Mental Status Exam (MMSE), the Instrumental Activities of Daily Living Scale (IADL), the Geriatric Depression Scale (GDS), and the Timed Get Up and Go Test (TUG). Out of a pool of 300 subjects recruited by June 2005, we selected 47, who scored very high on the geriatric tests, for candidate gene re-sequencing. Average scores for these geriatric tests were: MMSE = 28.5, IADL = 22.3, GDS = 1.1, and TUG = 11.3. Forty-six of these sequenced subjects have all four grandparents of European ancestry; one is Southeast Asian. Genomic DNA was extracted from peripheral blood samples using the PureGene DNA isolation kit (Gentra Systems, MN) according to the manufacturer’s instructions.

### Conserved Nucleotide Sequences

Conserved nucleotide sequences (CNS) were identified by phylogenetic footprinting using the VISTA browser (http://pipeline.lbl.gov/cgi-bin/gateway2). CNS regions were selected in introns and within 15 kb up- and downstream of candidate genes. Genomic sequences of at least four organisms were aligned with the human reference genome. Available genomic sequences included chimpanzee, baboon, rhesus monkey, cow, dog, horse, opossum, mouse, rat, rabbit, chicken, frog, zebrafish, and fugu. Alignments with organisms that are in evolutionary terms either extremely close (chimpanzee, baboon) or not (fugu, zebrafish) were usually less informative. The selection criterion for CNS was a minimum of 70% conservation over at least 100 bp.

### Bidirectional Sequencing

PCR primers (see **Supplemental online Table S2**) were designed for 716 genomic regions, including 515 exons and 201 putative gene regulatory regions (CNS), which include 1500 bp upstream of the transcriptional start site of each gene. A total of ∼360 Kb of DNA sequence was PCR amplified per individual. Coding exons were amplified using primers designed in the intronic sequences flanking the exon boundaries to allow sequencing across all intron/exon junctions. The average amplicon size was 513 bp, the maximum 700 bp. Exons and CNS regions that span more that 700 bp were amplified in overlapping segments. Primers for 14 genes were designed manually for DNA sequences retrieved from the UCSC genome browser (hg18) using the program Primer3 [46]. Primers for the remainder were downloaded from the NCBI probes webpage (http://www.ncbi.nlm.nih.gov/sites/entrez?db=probe) and further supplemented with primers designed in-house for regions not covered. Forward and reverse primers incorporated the -21M13F (TGTAAAACGACGGCCAGT) or M13R (CAGGAAACAGCTATGAC) extensions, respectively, at their 5′ ends. PCR, sequencing reactions and sequence analysis procedures were carried out as described previously [47]. Briefly, PCR reactions were optimized for each individual primer pair using genomic test DNA and a temperature gradient (48–65°C). Standard PCR conditions were 15 s annealing time, 30 s extension time, and 35 cycles. A standard 10 ul (optimization) or 20 ul (sample) PCR reaction mix contained 1 mM MgSO_4_, 0.2 mM dNTPs, 0.5 uM of each primer, 0.0125 units Platinum Pfx Polymerase, 1× Enhancer solution, 1× Pfx amplification buffer (all from Invitrogen, CA, USA) and 10 ng genomic DNA. Primer pairs showing no product or high background amplification were re-tested at slightly different conditions (annealing time: 5 s–1 min; extension time: 10 s–1 min) or re-designed if necessary (∼5% of primers). The majority of primers worked at 60°C (±3°C). All PCR products, optimizations as well as sample PCRs, were checked on 2% agarose gels (SeaKem LE, Cambrex, ME, USA). For cycle sequencing, we used Big Dye Terminator Mix v3.1 (Applied Biosystems, Foster City, CA) at 0.33 µl of mix per reaction in a total volume of 4 µl with 50 cycles of amplification and ABI 3730 capillary sequencers. DNA sequence for 14 out of 24 genes was analyzed with Phred/Phrap/polyphred-5.02/polyphred-7/Consed 14 as described [47]. The remainder of genes was analyzed using Mutation Surveyor (Softgenetics, PA, USA). 5 genes were analyzed with both software tools to demonstrate no appreciable difference in the detection of sequence variants. In total we generated and analyzed 34.8 million base pairs. All variants were verified by at least two researchers.

### TagSNP selection

For all 24 candidate genes we inferred tagSNPs from our sequenced variants as well as from data available through the HapMap project. European variants for our candidate gene regions (±10 Kb) and a MAF ≥5% were obtained from the HapMap website (www.hapmap.org). For the gene re-sequencing data, we generally considered variants (SNPs and insertion/deletions) with MAF ≥2%; the 684 re-sequencing variants included 30 variants with a MAF <2%. The Tagger tagSNP selection algorithm implemented in Haploview (version 4.1, http://www.broad.mit.edu/node/443) was used for selecting tagSNPs. 3% of singleton variants (MAF = 1.1%) are exclusively observed in the Southeast Asian sample as opposed to over 30% in the European samples. These specific Southeast Asian variants are included in our report but were excluded from the tagSNP analysis.

## Supporting Information

Table S1 Variants discovered by candidate gene sequencing
(0.25 MB XLS)

Table S2 PCR primer information
(0.22 MB XLS)
